# Home administration of lanreotide Autogel® by patients with acromegaly, or their partners, is safe and effective

**DOI:** 10.1111/j.1365-2265.2007.03044.x

**Published:** 2008-03

**Authors:** J S Bevan, J Newell-Price, J A H Wass, S L Atkin, P M Bouloux, J Chapman, J R E Davis, T A Howlett, H S Randeva, P M Stewart, A Viswanath

**Affiliations:** * Department of Endocrinology, Aberdeen Royal Infirmary Foresterhill, Aberdeen, London, UK; † Endocrine Unit, Royal Hallamshire Hospital Sheffield, London, UK; ‡ Department of Endocrinology, Oxford Centre for Diabetes, Endocrinology and Metabolism, Churchill Hospital Oxford, London, UK; § Michael White Centre for Diabetes & Endocrinology, Hull Royal Infirmary, Hull, East Yorkshire Hampstead, London, UK; ¶ Department of Endocrinology, The Royal Free Hospital Hampstead, London, UK; ** Department of Endocrinology, Sunderland Royal Hospital Sunderland, UK; †† Department of Endocrinology, Manchester Royal Infirmary Manchester, UK; ‡‡ Department of Endocrinology, Leicester Royal Infirmary, Leicester Leicestershire, UK; §§ Department of Endocrinology, University Hospitals of Coventry & Warwickshire NHS Trust, Coventry Edgbaston, Birmingham, UK; ¶¶ Department of Medicine, Queen Elizabeth Hospital, Queen Elizabeth Medical Centre Edgbaston, Birmingham, UK

## Abstract

**Objective:**

The introduction of ready-to-use lanreotide Autogel® has presented the possibility of patients receiving their acromegaly treatment at home. The objective of this study was to assess the ability of patients (or their partners) to administer repeat, unsupervised, injections of lanreotide Autogel without compromising efficacy or safety.

**Design:**

Multicentre (10 UK regional endocrine centres), open-label, nonrandomised, controlled study. Patients elected either to receive/administer unsupervised home injections after injection technique training (Test group) or continued to receive injections from a healthcare professional (Control group). Patients received monthly injections of lanreotide Autogel® at their established dose. Effects were monitored for up to 40 weeks.

**Patients:**

Thirty patients (15 per treatment group) with acromegaly treated with a stable dose of lanreotide Autogel® (60, 90 or 120 mg) for ≥ 4 months before screening.

**Measurements:**

The main outcome measure was the proportion of patients/partners who successfully administered injections throughout the study.

**Results:**

All Test group patients/partners qualified to administer injections. Fourteen of 15 patients fulfilled all criteria for successful administration of unsupervised injections (95% confidence interval, 70%–99%). Fourteen of 15 Test and 14/15 Control patients maintained growth hormone and IGF-1 control. Local injection tolerability was good for both treatment groups, and safety profiles were similar. All Test group patients continued with unsupervised injections after the study.

**Conclusions:**

Patients with acromegaly or their partners were able to administer lanreotide Autogel® injections with no detrimental effect on efficacy and safety; therefore, unsupervised home injections are a viable alternative to healthcare professional injections for suitably motivated patients.

## Introduction

The introduction of somatostatin analogues was a major therapeutic advance in the management of acromegaly. Slow-release, long-acting formulations, administered by fortnightly or monthly injections, have been found to control plasma GH levels, normalize serum levels of IGF-1 and reduce clinical symptoms in over 50% of patients.^1^

Lanreotide Autogel® (Ipsen Pharma Biotech SAS, Signes, France) is a long-acting viscous aqueous formulation of lanreotide, which is supplied in ready-to-use prefilled syringes intended for deep subcutaneous injection. Lanreotide Autogel® provides consistent drug release and is effective in controlling the biochemical markers and symptoms of acromegaly when injected every 28 days^1^–^8^. Lanreotide Autogel® does not require reconstitution, a process which can be problematic and cause administration problems due to needle blockage by microparticles.

The availability of a ready-to-use preparation has raised the possibility of home administration by the patient or a trusted relative or friend (partner). This could be beneficial to patients in reducing the impact of the disease on their daily lives and also in improving acceptability of long-term therapy. In addition, if lanreotide Autogel® can be safely administered unsupervised, while maintaining disease control, this would reduce the clinic time required by these patients.

The aim of this study was to assess whether patients with acromegaly could successfully self-administer, or receive from their partners, monthly injections of lanreotide Autogel® without any loss of clinical efficacy or deterioration in the safety profile.

## Patients and methods

Prior to patient enrolment, the study was approved by the London Multicentre Research Ethics Committee and the Local Research Ethics Committee for each study centre. It was conducted in accordance with the Declaration of Helsinki and the International Conference on Harmonization – Good Clinical Practice. All patients, and their partners if applicable, gave written informed consent before entering the study.

### Patients

Thirty patients aged 18 years or older with a clinical diagnosis of acromegaly were to be included in this study. All patients must have been treated with a stable dose of lanreotide Autogel (60, 90 or 120 mg per month) for at least 4 months before entering the study. Study patients were required to have a mean serum GH level ≤ 10·0 µg/l within 4 weeks of the Baseline visit, and a life expectancy of at least 12 months at the time of screening. Patients were excluded if they had undergone pituitary surgery within 6 months or radiotherapy within 1 year of study screening, or if they were likely to require either of these interventions during the study period. Other exclusion criteria included ongoing treatment with a GH antagonist or somatostatin analogue other than lanreotide Autogel.

### Methods

This was a phase IV, multicentre, open-label, controlled study to assess the ability of suitably motivated patients with acromegaly, or their partners, to administer repeat injections of lanreotide Autogel without supervision from a healthcare professional (defined as an endocrinologist or endocrine nurse specialist).

Following screening (week –4) to assess patient eligibility, patients chose whether to enter the Test or the Control group at the Baseline visit (week 0). All patients were given a lanreotide Autogel injection by a healthcare professional at the Screening and Baseline visits. Control group patients continued to receive their lanreotide Autogel injections every 4 weeks from a healthcare professional throughout the study. After the Baseline visit, Control group patients received a further nine injections (weeks 4–36). Test group patients either administered the injections themselves or received their injections from a ‘partner’. ‘Partner’ was defined as any person whom the patient trusted and deemed responsible enough to administer their injections. Having observed the healthcare professional demonstrate the injection technique at Baseline, Test group patients (or their partners) performed between one and three supervised training injections at weeks 4, 8 and 12, as necessary. The ability of the patient/partner to perform the injections was evaluated by a healthcare professional after each supervised injection. Once the patients (or partners) were considered qualified to administer unsupervised injections, they were provided with supplies of study drug enabling six subsequent unsupervised injections to be administered at home once every 4 weeks.

All patients received lanreotide Autogel at the same dose they had been receiving at the time of entry into the study. Lanreotide Autogel was administered by deep subcutaneous injection into the upper external quadrant of the buttock (for healthcare professional and partner injections) or into the upper outer thigh (for self-injections).

The effects of treatment were monitored for a maximum of 40 weeks. Throughout the study, all patients were required to complete diary cards to record information about injections administered, tolerability and safety.

### Outcome measures

Mean GH, IGF-1 and drug (serum lanreotide) concentrations were assessed at Baseline, Interim visit, and Study Completion. These analyses were done using fasting samples collected before administration of the study medication (i.e. at trough lanreotide levels). Mean GH was calculated from between five and nine blood samples taken at 30-min intervals over a 4-h period in the morning.

Interim visits were conducted at week 20 for Control group patients and after three unsupervised injections for the Test group (week 20, 24 or 28, depending on the number of training injections required). Study Completion visits were conducted at week 40 for Control group patients and after six unsupervised injections (week 32, 36 or 40) for the Test group.

The primary efficacy endpoint was the proportion of patients (or partners) who successfully administered home injections of lanreotide Autogel throughout the study, as assessed using the following criteria:

- The patient (or partner) was declared qualified by a healthcare professional to administer unsupervised injections after a maximum of three training injections;
- The patient had received adequate treatment throughout the study as assessed by the healthcare professional at the Study Completion visit;
- The patient's disease control (assessed by evaluating plasma GH and IGF-1 levels) was maintained.

Secondary endpoints included:

- The proportion of patients whose GH ‘control band’ (i.e. mean GH either above or below an arbitrary level of 5 µg/l) and whose age-related IGF-1 level ‘control band’ (low/normal or elevated) at the Interim and Study Completion visits was the same as or improved from Baseline;
- The change in mean GH, IGF-1 and serum lanreotide levels from Baseline to the Interim and Study Completion visits;
- Patient assessment of injection site tolerability;
- Patient (or partner) and healthcare professional evaluation of unsupervised injections.

Safety measurements were made throughout the study and included assessments of adverse events (AEs), clinical laboratory tests, physical examinations and vital signs.

### Assay methods

All laboratory samples were analysed by a central laboratory.

### GH

Serum GH levels were measured using an Immulite 1000 kit (solid-phase, two-site chemiluminescent immunometric assay) according to the manufacturer's instructions. The assay has an analytical sensitivity of up to 0·1 µg/l and a broad working range of 0·2–40 µg/l. Conversion: µg/l × 2·6 = mIU/l (WHO 1st IS 80/505).

### IGF-1

Serum IGF-1 levels were measured using an Immulite 1000 kit (solid-phase chemiluminescent immunometric assay) according to the manufacturer's instructions. The analytical sensitivity of the assay is 0·02 µg/l.

### Drug concentration

Serum lanreotide levels were measured using a validated, in house, Ipsen radioimmunoassay method. The assay has a lower detection limit of 0·078 µg/l and an accepted working range of 0·078–0·480 µg/l. The result of any sample was accepted if the coefficient of variation of the duplicate was < 15%.

### Statistical methods

Given the exploratory nature of the study, no formal statistical analysis was performed. Summary statistics were calculated for the primary and secondary endpoints and reported with their two-sided 95% confidence intervals (CIs), calculated using the Wilson binomial method. Efficacy analyses used the intention to treat population (ITT; patients who received at least one dose of study medication and who provided data for at least one efficacy variable) and the safety analyses used the safety population (patients who received at least one dose of study medication and who provided any follow-up data for efficacy or safety variables). The primary endpoint was also assessed using the per protocol (PP) population, which included all patients who completed the trial in accordance with the study protocol.

## Results

### Patients studied

Thirty patients aged 29–86 years (43% female, *n* = 13) were enrolled, received at least one dose of study medication and completed the study (Test, *n* = 15; Control, *n* = 15). The baseline demographic characteristics of the study population are summarized in Table 1. Test group patients were younger than those in the Control group (*P* = 0·0001) and were more likely to have undergone previous pituitary surgery (*P* < 0·0001) and radiotherapy (not significant). The mean length of prior treatment with lanreotide Autogel (*P* = 0·0092) and other somatostatin analogues (not significant) was longer for Control than Test patients.

**Table 1:** Demographic and baseline characteristics

Variable	Control group (*n* = 15)	Test group (*n* = 15)	*P*-value
Mean (SD) age, years	69·2 (8·1)	49·7 (14·0)	0·0001
Mean (SD) weight, kg	94·4 (19·9)	85·8 (14·8)	NS
Gender			
Male, *n* (%)	9 (60)	8 (53)	NS
Female, *n* (%)	6 (40)	7 (47)	NS
Mean (SD) time since diagnosis, months	116·8 (64·2)	109·4 (91·6)	NS
Pituitary surgery, *n* (%)	4 (27)	14 (93)	*<* 0·0001
Radiotherapy, *n* (%)	5 (33)	7 (47)	NS
Mean (SD) length of treatment with lanreotide Autogel, months	29·7 (19·5)	13·9 (7·7)	0·0092
Mean (SD) length of treatment with other somatostatin analogue(s), months	64·4 (27·4)	55·5 (43·5)	NS

SD, standard deviation; NS, not significant.

Of the 15 Test group patients, 12 chose to self-administer and three had their injections administered by their partner. All Test group patients (or their partners) were declared qualified to administer unsupervised injections after training; 14 patients required only one injection training session and although the remaining patient was considered by the healthcare professional to be qualified after the first training session, the patient requested a further supervised injection.

In the Test group, five patients received the 60-mg dose of lanreotide Autogel, five patients received the 90-mg dose and five received the 120-mg dose. In the Control group, 10 patients received the 60-mg dose, three patients received the 90-mg dose, and two received the 120-mg dose.

There were three major protocol violations. In the Control group, one patient missed the week 16 and week 24 injections, and therefore the other visits were later than scheduled, and one patient missed the week 16 injection, and therefore their Interim visit was early. In the Test group, one patient administered an unscheduled seventh unsupervised injection a day before the Study Completion visit, which was therefore rescheduled for a month later. Patients with major protocol violations were excluded from the PP population.

### Efficacy

#### Primary endpoint

Fourteen of the 15 Test group patients (93%) fulfilled all of the criteria for successful administration of unsupervised injections (ITT population: 95% CI 70–99%; Table 2); the results of the PP population supported this (93%; 95% CI 69–99%). One patient did not meet the defined criteria for successful treatment as their mean GH level increased from < 5 µg/l at Baseline to > 5 µg/l at Study Completion (see footnote to Table 2). This patient had been established on treatment with 60 mg lanreotide Autogel for 4 months before the study.

**Table 2:** Overall outcomes

Criteria	Control group (*n* = 15) Number of patients (%)	Test group (*n* = 15) Number of patients (%)
Qualified after training	N/A	15 (100)
Received adequate treatment	15 (100)	15 (100)
GH level maintained	15 (100)	14 (93)*
IGF-1 level maintained	14 (93)†	15 (100)
Overall treatment success	N/A	14 (93)

N/A, not applicable.

* One patient (60-mg dose) had a GH level of 5·0 µg/l at Screening, 2·0 µg/l at Baseline, 3·3 µg/l at Interim assessment and 6·7 µg/l at Study Completion.

† One patient (60-mg dose) had an IGF-1 level of 185 µg/l at Screening, 181 µg/l at Baseline, 194 µg/l at Interim assessment and 241 µg/l at Completion.

#### Secondary endpoints

**Fig. 1:**
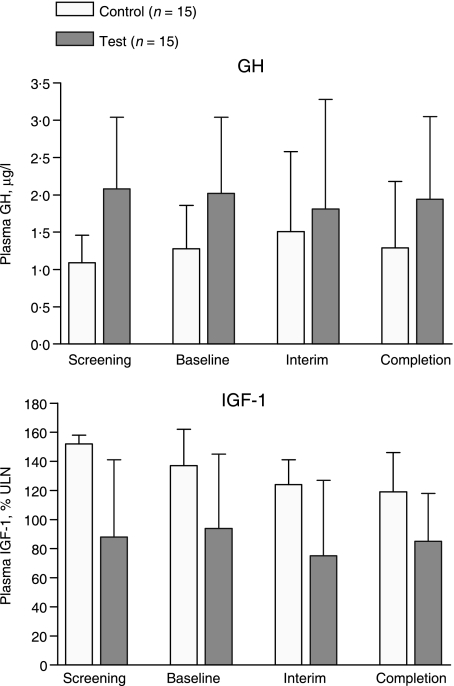
Median serum (and upper quartile) GH and IGF-1 levels throughout the study. GH, growth hormone; IGF-1, insulin-like growth factor-1.

**Fig. 2:**
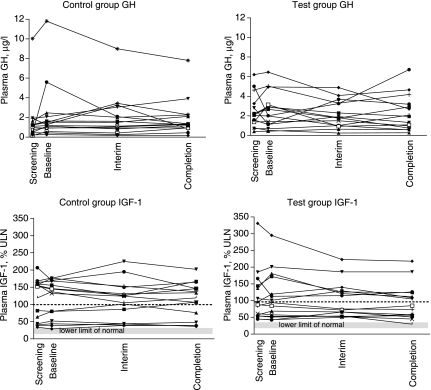
Individual GH and IGF-1 levels throughout the study. ULN, upper limit of normal.

All patients in the Test and Control groups maintained GH control (i.e. all patients were in the same or an improved GH control band) at the Interim visit compared with Baseline. Fourteen out of the 15 Test group patients (93%) and all 15 Control group patients maintained GH control at Study Completion compared with Baseline (treatment difference Test *vs.* Control, –7%; 95% CI –30 to –14%; Table 2, Fig. 1). In one Test group patient, the mean GH level increased from 2·0 µg/l at Baseline to 6·7 µg/l at Study Completion (patient previously mentioned in connection with the primary endpoint; Table 2, Fig. 2). For both treatment groups, there were no notable changes in mean and median GH levels throughout the study (Table 3, Fig. 1).

**Table 3:** Summary of GH, IGF-1 and serum lanreotide levels throughout the study (ITT population)

Variable	Control group (*n* = 15)	Test group (*n* = 15)	Median difference between treatment groups (95% CI)
**GH level (µg/l)**			
*Baseline*			
Mean (SD)	2·22 (2·94)	2·49 (1·81)	
Median	1·28	2·02	
*Study Completion*			
Mean (SD)	1·88 (1·87)	2·27 (1·82)	
Median	1·29	1·94	
*Change from Baseline*			
Mean (SD)	–0·34 (1·63)	–0·23 (1·79)	
Median	0·02	–0·23	–0·28 (–1·36 to –0·39)
**IGF-1 (% ULN)**			
*Baseline*			
Mean (SD)	115 (55)	112 (72)	
Median	137	94	
*Study Completion*			
Mean (SD)	111 (53)	96 (53)	
Median	119	85	
*Change from Baseline*			
Mean (SD)	–4 (17)	–16 (28)	
Median	–5	–11	–8 (–24 to –9)
**Serum lanreotide (µg/l)**			
*Baseline*			
Mean (SD)	3·26 (1·52)	2·60 (1·13)	
Median	2·83	2·62	
*Study Completion*			
Mean (SD)	2·73 (1·42)	2·89 (1·20)	
Median	2·39	2·55	
*Change from Baseline*			
Mean (SD)	–0·76 (1·83)	0·16 (1·11)	
Median	–0·71	0·32	0·89 (–0·21 to –1·74)

ITT, intention to treat; SD, standard deviation; ULN, upper limit of normal; 95% CI, 95% confidence interval.

All 15 Test group patients and 14 out of 15 Control group patients maintained IGF-1 control (i.e. were in the same or an improved IGF-1 control band) at the Interim visit compared with Baseline. All Test group patients and 14 out of 15 Control group patients maintained IGF-1 control at Study Completion compared with Baseline (treatment difference Test *vs.* Control, 7%; 95% CI –14 to –30%). One Control group patient had worsened IGF-1 levels at the Interim visit (normal to elevated), but this had returned to normal on Study Completion. Another Control group patient moved from normal (181 µg/l) to elevated (241 µg/l) on Study Completion (Table 3, Fig. 2). This patient had been established on treatment with 60 mg lanreotide Autogel 26 months before the study. For both treatment groups, there was a decrease in mean (and median) IGF-1 levels from Baseline to Study Completion. The decrease was more notable in the Test group (Table 3, Fig. 1).

There were no notable changes in serum lanreotide concentrations in either the Test or Control group patients from Baseline to the Interim and Study Completion visits (Table 3).

Local injection site tolerability was good for both treatment groups with the majority of patients experiencing either none or only mild pain, redness or swelling. Each patient's perception of the pain associated with the injection remained fairly constant throughout the study. Within the Test group, the transfer to self- (or partner) injections from those administered by a healthcare professional did not affect the frequency or intensity of pain. There was a small increase in the number of reports of mild redness or swelling during the self- (or partner) administration period of the study, but these were usually mild. The time taken to administer the injections was comparable between treatment groups, mostly taking no more than 5 min to administer.

### Safety

The most common AEs were gastrointestinal, for example, diarrhoea, upper abdominal pain, nausea and constipation. No patients withdrew from the study because of AEs. There were no clinically relevant changes or differences between treatment groups in any clinical laboratory tests, vital signs or physical examination results.

## Discussion

This study investigated whether patients with acromegaly could be successfully trained to self-administer, or receive from a partner, unsupervised monthly injections of lanreotide Autogel. All patients (or partners) were successfully trained to administer unsupervised injections. At the end of the study, 14 of the 15 patients met all criteria for successful administration of unsupervised injections, which suggests that unsupervised self- or partner depot injections are a feasible treatment option for some patients with acromegaly. One patient had fluctuating GH levels throughout the study and slightly elevated mean GH levels on Study Completion compared with Baseline. This patient had been on a 60-mg dose for only 4 months before the start of the study, and erratic GH control may have been due to suboptimal dosing rather than problems with lanreotide Autogel administration. Both treatment groups had, overall, well-maintained disease control, as assessed by the maintenance of GH and IGF-1 levels. There was no loss of efficacy in patients who received unsupervised injections compared with patients who continued to receive injections from a healthcare professional. In addition, there were no notable changes in serum lanreotide concentrations throughout the study in either study group.

The injections were well tolerated by patients in both treatment groups; there was no increase in the frequency or severity of pain when patients moved to self- (or partner) injections from receiving them from a healthcare professional. The small increase in reports of redness and swelling in Test group patients was likely to reflect the increased visibility of the injection site (thigh *vs.* buttock) rather than a true increase in the frequency of redness and swelling.

There are a number of advantages to carrying out unsupervised injections; for example, flexibility is increased as the patient can receive their injections at home or away from home, for example, on holiday or at work. In addition, the time spent travelling to and attending a clinic for their treatment is greatly reduced. Lanreotide Autogel also lends itself to self- or partner injection as it is supplied in a ready-to-use syringe with a small injection volume. The fact that lanreotide Autogel does not require reconstitution prior to injection makes this preparation particularly suitable for self-administration.

The design of this study meant that patients were not randomized to treatment; patients chose which method of treatment they were willing to receive. This method will have inevitably introduced bias and resulted in some differences in the baseline characteristics of the groups. However, unsupervised injections would not appeal to all patients, and therefore this reflects the choice that patients make in a normal clinical setting. We found that the patients likely to consider unsupervised injections are more likely to be the younger patients of working age who were seeking to gain increased independence from medical care. The 6-month duration of self-/partner injections in this study was considered an appropriate length of time for changes in GH and IGF-1 levels to be observed if the injections were not being administered correctly.

This is the first study showing that lanreotide Autogel can be successfully self- or partner administered in an unsupervised environment without affecting overall disease control. It also supports previous studies using lanreotide Autogel, confirming that it is an effective and well-tolerated treatment.^1^–^7^ Although not specifically assessed in this study, self- (or partner) injections reduce the number of clinic appointments required by patients and therefore would be expected to have cost benefits for healthcare providers.
